# Some characteristics of clinical sequelae of COVID‐19 survivors from Wuhan, China: A multi‐center longitudinal study

**DOI:** 10.1111/irv.12943

**Published:** 2021-11-19

**Authors:** Xian Chen, Ying Li, Tong‐Ren Shao, Ling‐Li Yang, Si‐Jing Li, Xiu‐Juan Wang, Ao Li, Yin‐Yu Wu, Xue‐Fei Liu, Chun‐Mei Liu, Yu‐Hui Liu, Fan Zeng, Yuan Cen

**Affiliations:** ^1^ Department of Anesthesiology, Daping Hospital Army Medical University Chongqing China; ^2^ Department of Ophthalmology, Daping Hospital Army Medical University Chongqing China; ^3^ Department of Medical Education, Daping Hospital Army Medical University Chongqing China; ^4^ Department of Orthopedics, Daping Hospital Army Medical University Chongqing China; ^5^ Department of Neurology and Center for Clinical Neuroscience, Daping Hospital Army Medical University Chongqing China

**Keywords:** clinical sequelae, COVID‐19, prevalence, risk factor, survivors

## Abstract

**Background:**

The pandemic of COVID‐19 has a persistent impact on global health, yet its sequelae need to be addressed at a wide scale around the globe. This study aims to investigate the characteristics, prevalence, and risk factors for mid‐term (>6 months) clinical sequelae in a cohort of COVID‐19 survivors.

**Methods:**

Totally 715 COVID‐19 survivors discharged before April 1, 2020, from three medical centers in Wuhan, China, were included. The longitudinal study was conducted by telephone interviews based on a questionnaire including the clinical sequelae of general, respiratory, and cardiovascular systems. Demographics and some characteristics of clinical sequelae of the survivors were recorded and analyzed. Multivariate logistic regression analysis was applied to explore the risk factors for the sequelae.

**Results:**

The median time interval from discharge to telephone interview was 225.0 days. The COVID‐19 survivors' median ages were 69 years, and 51.3% were male. Among them, 29.9% had at least one clinical sequela. There were 19.2%, 22.7%, and 5.0% of the survivors reporting fatigue, respiratory symptoms, and cardiovascular symptoms, respectively. Comorbidities, disease severity, the application of mechanical ventilation and high‐flow oxygen therapy, and the history of re‐admission were associated with the presence of clinical sequelae.

**Conclusions:**

Our study provides further evidence for the prevalence and characteristics of clinical sequelae of COVID‐19 survivors, suggesting long‐term monitoring and management is needed for their full recovery.

## 1 INTRODUCTION

By July 23, 2021, there have been 192,284,207 confirmed cases of coronavirus disease 2019 (COVID‐19), including 4,136,518 deaths worldwide (https://covid19.who.int/). The pandemic of this infectious disease has a persistent impact on global health. A recent study found that among patients who recovered from COVID‐19 and were discharged from the hospital after approximately 1 month, 87.4% reported persistence of at least one symptom, particularly fatigue and dyspnea.^1^ Previously, patients who recovered from severe acute respiratory syndrome (SARS) had radiological, functional, and psychological sequelae, both at short‐term and long‐term clinical follow‐up.^2^, ^3^ Since the clinical features of COVID‐19 and SARS are similar, and their pathogens both belong to beta coronaviruses,^4^ COVID‐19 survivors may also suffer from sequelae in different systems.

Clarifying the clinical sequelae of COVID‐19 is crucial for its monitoring and management in the long run. Up till now, there have been a few relevant studies, and the follow‐up time ranged from 1 to 6 months.^5^, ^6^, ^7^, ^8^, ^9^, ^10^ Being the first country stricken by COVID‐19 since December 2019, China was supposed to have a large number of COVID‐19 survivors with more prolonged convalescence than the others.^11^, ^12^ Therefore, we conducted this multi‐center longitudinal questionnaire‐based study to investigate the characteristics, prevalence, and risk factors for mid‐term clinical sequelae in a cohort of COVID‐19 survivors in China.

## 2 MATERIALS AND METHODS

### 2.1 Study design and participants

This study included participants from three cohorts of inpatients diagnosed with COVID‐19 according to WHO interim guidance^13^ and discharged from Huoshenshan Hospital, General Hospital of the Central Theatre Command of the People's Liberation Army, and mobile cabin hospitals in Wuhan, China. The four categories of disease severity, including mild, moderate, severe, and critical, were determined according to *Diagnosis and Treatment Protocol for Novel Coronavirus Pneumonia (6th edition)*, which was released by the National Health Commission of China (http://www.nhc.gov.cn/xcs/zhengcwj/202002/8334a8326dd94d329df351d7da8aefc2.shtml). Mild cases were defined as mild symptoms without abnormalities on chest CT. Moderate cases were defined as mild symptoms with abnormalities on chest CT. Severe cases were defined as either: (a) respiratory rate ≥30 breaths/min, or (b) oxygen saturation ≤93%, or (c) PaO_2_/FiO_2_ ratio ≤300 mmHg. Critical cases were defined as those including one criterion as follows: shock, respiratory failure requiring mechanical ventilation, and organ failure requiring admission to ICU. Patients discharged before April 1, 2020, were considered as appropriate candidates for observing their clinical sequelae in mid‐term convalescence from COVID‐19, and telephone interview follow‐ups were conducted from October 1 to November 1, 2020. Therefore, the interval time from discharge to the telephone follow‐up initiation was more than 6 months. Patients with severe and complex comorbidities were excluded, including malignant tumors, mental disorders, severe anemia, cirrhosis, and chronic renal failure. The study was approved by the institutional board of each participating site. Verbal consents were obtained from all participants.

### 2.2 Telephone interviews and data collection

Based on the previously reported clinical characteristics of COVID‐19 patients and studies on the sequelae of SARS,^1^, ^2^, ^12^ we conducted the telephone interviews according to a formal questionnaire about the clinical sequelae mainly on three aspects: (1) general symptoms: referring to fatigue or physical decline compared with the status before developing COVID‐19, and the other symptoms not included in the questionnaire while self‐reported by the interviewees; (2) respiratory symptoms: including cough, sputum, exertional or resting dyspnea, and chest tightness; (3) cardiovascular symptoms: including palpitation, orthopnea, and lower limb edema. All telephone interviews were conducted by experienced nurses by mobile phone. All participants were instructed that only the remaining or newly occurring symptoms after developing COVID‐19 were needed to report at the beginning of the interview, to avoid recording the possible long‐standing symptoms unrelated to COVID‐19 as much as possible. The demographics, categories of disease severity, the medical history including hypertension, diabetes, hyperlipidemia, stroke, and coronary heart disease as well as the history of the application of high‐flow oxygen, admission to ICU, mechanical ventilation during hospitalization, and the history of re‐admission to the hospital after discharge were also recorded. All data were carefully reviewed by the major investigators of this study.

### 2.3 Data analysis

Continuous variables were shown as medians (interquartile ranges, IQR). Categorical variables were summarized as the counts and percentages for each category. To avoid selection bias, the differences in age, sex, disease severity, and comorbidity between the study population and the population lost to follow‐up were compared using *t* test and chi‐square test as appropriate. The univariate analysis of risk factors for the clinical sequelae was done by chi‐square test. To further explore the risk factors associated with the clinical sequelae, multivariate logistic regression analysis was applied with the forward stepwise (conditional) method. The multicollinearity of all covariates was examined using the collinearity analysis and the variance inflation factor. All analyses were conducted with PASW version 18.0 for windows (SPSS, Inc., Chicago, IL). *P* < 0.05 was considered statistically significant.

## 3 RESULTS

### 3.1 Demographics and characteristics of the participants

A total of 964 adult COVID‐19 patients discharged from three hospitals in Wuhan from our previous study were firstly screened.^14^ Of these, 13 patients (1.4% of the total survivors) were excluded because of chronic renal failure who needed routine hemodialysis (n = 11) or cirrhosis due to hepatitis B infection (n = 2), considering they were likely to report unspecific symptoms which may confound the results. Then 951 patients discharged before April 1, 2020, were considered as the candidate participants. During the telephone follow‐ups, 236 cases did not complete the interview, of whom 54 could not be contacted by the recorded telephone numbers and 182 refused to participate in our study. Finally, 715 COVID‐19 survivors accomplished the complete telephone interview follow‐up. The flow chart of the study sample is shown in Figure S1. There were no differences in age, sex, disease severity, and comorbidity between the study population (n = 715) and the population lost to follow‐up (n = 236) (see Table S1).

The time interval from discharge to telephone interview was 225.0 days (IQR 222.0–228.0). The survivors' median ages were 69 years (IQR 67–73), and 51.3% (367/715) were male. The detailed demographics, categories of disease severity, medical history including hypertension, diabetes, hyperlipidemia, stroke, and coronary heart disease, as well as the history of the application of high‐flow oxygen or mechanical ventilation, admission to ICU during hospitalization, and re‐admission to the hospital after discharge are shown in Table 1.

**TABLE 1:** Demographic and clinical features of the studied COVID‐19 survivors

Variables	COVID‐19 survivors (n = 715)
Interval time^a^ (days), median (IQR)	225.0 (222.0–228.0)
Age (years), median (IQR)	69 (67–73)
55–64	100 (14.0%)
65–74	467 (65.3%)
>74	148 (20.7%)
Male, n (%)	367 (51.3%)
Disease severity, n (%)	
Mild	541 (75.7%)
Moderate	95 (13.3%)
Severe	48 (6.7%)
Critical	31 (4.3%)
Comorbidity, n (%)	
Hypertension	277 (38.7%)
Diabetes	165 (23.1%)
Hyperlipidemia	83 (11.6%)
Stroke	31 (4.3%)
Coronary heart disease	77 (10.8%)
Treatment during hospitalization, n (%)	
High‐flow oxygen therapy	198 (27.7%)
Mechanical ventilation	62 (8.7%)
Admission to ICU	45 (6.3%)
Re‐admission after discharge, n (%)	48 (6.7%)
Positive RT‐PCR test results after discharge	41 (5.7%)
Other reasons	7 (1.0%)

*Note*: Data are median (IQR), n (%), where n is the total number of patients with available data.

Abbreviations: COVID‐19, coronavirus disease 2019; IQR, interquartile range.

^a^ Interval time, the time interval from discharge to telephone interview.

### 3.2 Characteristics and prevalence of clinical sequelae

Based on the results, 29.9% (214/715) of the COVID‐19 survivors had at least one clinical sequela included in the questionnaire. There were 137 (19.2%, 137/715) cases reporting fatigue or physical decline. The other symptoms not included in the questionnaire and self‐reported by the participants were throat discomfort (0.6%, 4/715), chest pain (0.8%, 6/715), myalgia (0.7%, 5/715), and others (2.0%, 14/715, including alopecia, lack of appetite, weight loss, etc.).

A total of 162 (22.7%) survivors had one or more respiratory symptoms, with exertional or resting dyspnea the most common one (11.9%, 85/715). The prevalence of cough, sputum, and chest tightness was 11.5% (82/715), 4.9% (35/715), and 6.9% (49/715), respectively. As for cardiovascular symptoms, 5.0% (36/715) of the survivors reported at least one clinical sequela. Among them, palpitation (3.8%, 27/715) was the most reported. The prevalence of orthopnea and lower limb edema was 0.6% (4/715) and 1.0% (7/715), respectively. The characteristics and prevalence of mid‐term clinical sequelae of COVID‐19 survivors are shown in Table 2.

**TABLE 2:** Characteristics and prevalence of mid‐term (more than 6 months after discharge) clinical sequelae of the COVID‐19 survivors

Clinical sequelae	COVID‐19 survivors (n = 715)
General symptoms, n (%)	159 (22.2%)
Fatigue	137 (19.2%)
Others	29 (4.1%)
Respiratory symptoms, n (%)	162 (22.7%)
Cough	82 (11.5%)
Sputum	35 (4.9%)
Exertional or resting dyspnea	85 (11.9%)
Chest tightness	49 (6.9%)
Cardiovascular symptoms, n (%)	36 (5.0%)
Palpitation	27 (3.8%)
Orthopnea	4 (0.6%)
Lower limb edema	7 (1.0%)

*Note*: Data are n (%), where n is the total number of patients with available data.

Abbreviation: COVID‐19, coronavirus disease 2019.

### 3.3 Risk factors for clinical sequelae of the COVID‐19 survivors

The results of univariate analysis of risk factors for the common clinical sequelae, including fatigue, respiratory and cardiovascular symptoms, are shown in Table S2. And the risk factors were further analyzed via multivariate logistic regression. The collinearity analysis showed no multicollinearity of all covariates, including age, sex, co‐morbidities, disease severity, the application of high‐flow oxygen, admission to ICU, mechanical ventilation during hospitalization, and the history of re‐admission after discharge (see Table S3). Therefore, they were all included in the regression analysis. It is shown in Table 3 that disease severity (moderate vs. mild, OR = 2.14, 95% CI 1.28–3.60; severe vs. mild, OR = 2.54, 95% CI 1.28–5.02), hypertension (OR = 1.59, 95% CI 1.07–2.37), hyperlipidemia (OR = 1.76, 95% CI 1.01–3.05), the history of re‐admission (OR = 4.52, 95% CI 2.29–8.92), and the application of mechanical ventilation during hospitalization (OR = 4.53, 95% CI 2.10–9.77) were associated with the risk of respiratory sequelae. Disease severity (moderate vs. mild, OR = 3.19, 95% CI 1.30–7.82; severe vs. mild, OR = 13.43, 95% CI 4.09–44.09; critical vs. mild, OR = 17.29, 95% CI 4.77–62.66), the history of re‐admission (OR = 3.38, 95% CI 1.26–9.10), and the application of high‐flow oxygen therapy (OR = 0.22, 95% CI 0.07–0.67) during hospitalization were associated with the risk of cardiovascular sequelae. Fatigue was associated with hypertension (OR = 1.65, 95% CI 1.10–2.47), the history of re‐admission (OR = 3.41, 95% CI 1.77–6.59), and the application of mechanical ventilation (OR = 5.52, 95% CI 3.11–9.81).

**TABLE 3:** Multivariate logistic regression analysis of risk factors for common clinical sequelae of COVID‐19 survivors

Clinical sequelae	Risk factors	OR	OR 95% CI	*P* values
Fatigue	Mechanical ventilation	5.52	3.11–9.81	<0.001
	Re‐admission after discharge	3.41	1.77–6.59	<0.001
	Hypertension	1.65	1.10–2.47	0.016
Respiratory symptoms	Disease severity			
	Moderate vs. Mild	2.14	1.28–3.60	0.004
	Severe vs. Mild	2.54	1.28–5.02	0.008
	Mechanical ventilation	4.53	2.10–9.77	<0.001
	Re‐admission after discharge	4.52	2.29–8.92	<0.001
	Hypertension	1.59	1.07–2.37	0.023
	Hyperlipidemia	1.76	1.01–3.05	0.046
Cardiovascular symptoms	Disease severity			
	Moderate vs. Mild	3.19	1.30–7.82	0.011
	Severe vs. Mild	13.43	4.09–44.09	<0.001
	Critical vs. Mild	17.29	4.77–62.66	<0.001
	High‐flow oxygen therapy	0.22	0.07–0.67	0.007
	Re‐admission after discharge	3.38	1.26–9.10	0.016

Abbreviations: CI, confidence interval; COVID‐19, coronavirus disease 2019; OR, odds ratio.

## 4 DISCUSSION

To the best of our knowledge, the follow‐up time of this study is by far the longest, with a median time of 225.0 days, among the studies on clinical sequelae of COVID‐19 survivors. According to our findings, COVID‐19 survivors are prone to suffer from clinical sequelae, including general, respiratory and cardiovascular symptoms, suggesting that clinical sequelae are common in their convalescence, even when cured and discharged.

Among the clinical sequelae included in the questionnaire, fatigue and respiratory symptoms were the most commonly reported, consistent with previous studies.^1^, ^5^, ^6^, ^7^, ^8^ The application of mechanical ventilation was associated with the risk of fatigue and respiratory sequelae. A possible explanation was that the pulmonary function of COVID‐19 patients who needed mechanical ventilation during hospitalization was usually worse than those who did not. Therefore, the recovery of pulmonary function for these patients is supposed to be longer and more difficult.^10^ This finding was supported by the results of a recent study showing that hospitalized patients with severe COVID‐19, who did not require mechanical ventilation, are unlikely to develop long‐term pulmonary impairments, thromboembolic complications, or cardiac impairments.^6^ The impact of ventilator‐induced lung injury on COVID‐19 survivors should also be considered.^6^, ^15^ Respiratory function examinations are warranted to assess their pulmonary function decline. The comorbidity of hypertension was another risk factor for the presence of fatigue and respiratory sequelae. In a recent study including over 44,000 patients with COVID‐19, hypertension, chronic respiratory disease, diabetes mellitus, cardiovascular disease, and cancer emerged as the most common comorbidities.^16^ Besides, hypertension was rendered to be a risk factor for disease progression and unfavorable outcomes.^14^, ^17^ Our study further provided evidence that hypertension could also be the risk factor for clinical sequelae. Angiotensin‐converting enzyme type 2 (ACE2) may link hypertension and disease susceptibility, disease progression, and possibly clinical sequelae of COVID‐19,^18^, ^19^ yet the underlying mechanism needs to be further explored.

It has been well documented that cardiac injury is a common condition among hospitalized COVID‐19 patients.^20^, ^21^ COVID‐19 patients with cardiac injury and pre‐existing cardiovascular disease are also associated with a higher risk of admission to ICU and in‐hospital mortality.^20^, ^22^ Therefore, the cardiovascular sequelae of COVID‐19 ought to be monitored. According to our findings, only 5% of COVID‐19 survivors had at least one cardiovascular symptom, which was much lower than the prevalence of respiratory sequelae. Interestingly, the application of high‐flow oxygen therapy during hospitalization seems to show a protective effect on cardiovascular sequelae. The high‐flow oxygen therapy has been proved to be beneficial for the clinical outcome of cardiac surgical patients and patients with acute heart failure^23^, ^24^; whether it is useful for the recovery of COVID‐19 needs to be further validated. The history of re‐admission after discharge was associated with a higher risk of all three clinical sequelae. Among the 48 patients who had a history of re‐admission, 41 were attributed to the reason that their RT‐PCR test results were positive once again when they finished the 14–28 days of quarantine after discharge. Although the “re‐positive” RT‐PCR result might be false‐positive, the impaired virus‐neutralizing ability, prolonged time of lung damage, and virus clearance among those patients may increase the prevalence of clinical sequelae.^17^, ^25^, ^26^

One study found that in 143 patients who had recovered from COVID‐19, 87.4% reported persistence of at least one symptom, particularly fatigue (53.1%) and dyspnea (43.4%) at approximately 36 days follow‐up since discharge.^1^ Another follow‐up study conducted 6 weeks after discharge showed that among the 33 severe COVID‐19 patients, 11 patients (33%) had dyspnea, 11 (33%) had cough, and 15 (45%) suffered from fatigue.^6^ In a recent study with a median time of 97.0 days (IQR 95.0–102.0) from discharge to the first follow‐up, the prevalence of clinical sequelae among 538 survivors was reported to be 28.3% for physical decline/fatigue (n = 152), 39.0% for respiratory symptoms (n = 210), and 13.0% for cardiovascular‐related symptoms (n = 70).^5^ Meanwhile, the results of our study showed that only 137 (19.2%) survivors had fatigue/physical decline, 162 (22.7%) had one or more respiratory symptoms, and 36 (5.0%) had at least one cardiovascular symptom. Based on these results, the prevalence of clinical sequelae of COVID‐19 tends to decrease over time. According to the findings of a 4‐year follow‐up study on 233 SARS survivors, 40.3% of them still reported a chronic fatigue problem.^3^ This phenomenon can be explained in several ways. First, the case‐fatality rate (CFR) of COVID‐19 is reported to be 2.3%, which is much lower than those of SARS (9.6%) and the Middle East respiratory syndrome (MERS, 34.4%),^16^ which indicates that the toxicity of SARS‐COV‐2 might be milder than SARS‐COV and MERS‐COV, and its damage to the respiratory and other systems may not be profound. This is supported by the evidence that, unlike SARS, the hospitalized patients with mild‐to‐moderate COVID‐19 were not at risk of developing pulmonary fibrosis.^27^ Consistently, in a 4‐month followed‐up study for 25 children convalescing from COVID‐19, mid‐term sequelae were also rarely observed.^28^ Second, both infected and non‐infected populations are vulnerable to anxiety, depression, and other psychological disorders during the pandemic.^29^, ^30^ It could not be excluded that at the early stage of convalescence, COVID‐19 survivors may experience certain mood disorders causing exaggerated self‐reported symptoms. Third, it is possible that the differences between these studies, including the different characteristics of the study population, disease severity, co‐morbidities, and definitions of sequelae, may lead to the apparent decline in the prevalence of clinical sequelae.

There are several limitations of this study. First, the questionnaire was designed by the major investigators of this study based on the common symptoms of COVID‐19 patients as well as the findings of previous relevant studies,^1^, ^2^, ^12^ which was not validated prior to this study. Second, because the study was conducted by telephone interviews, the related pathological situation of each COVID‐19 survivor could not be obtained. Therefore, there was a potential bias concerning the prevalence of clinical sequelae. This was also why we did not include the neurological and psychological symptoms in the questionnaire, as these symptoms can hardly be comprehensively evaluated in the form of telephone interviews rather than physical examinations and neuropsychological scales performed by specialists. Third, without a non‐COVID‐19 comparison group, the reported clinical sequelae were possibly related to exacerbated co‐morbidities. Fourth, the sample size was still not large enough compared with the total population of COVID‐19 patients in China. Nevertheless, the proportions of mild and moderate (89% vs. 81%), severe (6.7% vs. 14%), and critical cases (4.3% vs. 5%) of COVID‐19 survivors in this study were considered comparable to the population of Chinese COVID‐19 patients^16^ at the time when these survivors were discharged. Thirteen patients with severe and complex comorbidities were excluded because they were likely to report unspecific symptoms confounding the results. However, this might conceal some sequelae. Further studies on the clinical sequelae with a longer follow‐up time are needed to shed light on the trajectory of the clinical outcome of COVID‐19.

## 5 CONCLUSION

Clinical sequelae of general, respiratory and cardiovascular symptoms are common in COVID‐19 survivors, of which fatigue, exertional or resting dyspnea, and palpitation were the most prominent in this study. Comorbidities, degrees of disease severity, the application of mechanical ventilation during hospitalization, and the history of re‐admission after discharge may be associated with these sequelae. Our study provides evidence for the characteristics and prevalence of mid‐term (>6 months) clinical sequelae in COVID‐19 survivors, indicating that long‐term monitoring and proactive management are needed for their full recovery.

## AUTHOR CONTRIBUTIONS

**Xian Chen:** Data curation; investigation; validation. **Ying Li:** Data curation; investigation; validation. **Tong‐Ren Shao:** Data curation; formal analysis; software; validation. **Ling‐Li Yang:** Data curation; investigation. **Si‐Jing Li:** Data curation; investigation. **Xiu‐Juan Wang:** Data curation; investigation. **Ao Li:** Data curation; investigation. **Yin‐Yu Wu:** Data curation; investigation. **Xue‐Fei Liu:** Data curation; investigation. **Chun‐Mei Liu:** Data curation; investigation. **Yu‐Hui Liu:** Conceptualization; resources. **Fan Zeng:** Formal analysis; funding acquisition; project administration; visualization. **Yuan Cen:** Formal analysis; methodology; project administration; supervision.

### PEER REVIEW

The peer review history for this article is available at https://publons.com/publon/10.1111/irv.12943.

## Supporting information


**Table S1.** The comparison of characteristics between the study sample and cases lost to follow‐up
**Table S2.** The univariate analysis of risk factors for common clinical sequelae of COVID‐19 survivors.
**Table S3.** The collinearity analysis of all covariates in the multivariate logistic regression

**Figure S1.** The flow chart of the study sample

## Data Availability

The data that support the findings of this study are available from the corresponding author upon reasonable request.
