# Supplementary material for: Some characteristics of clinical sequelae of COVID‐19 survivors from Wuhan, China: A multi‐center longitudinal study
Source: Influenza Other Respir Viruses. 2021 Nov 19;16(3):395–401. doi: 10.1111/irv.12943 (PMC8652839; doi:10.1111/irv.12943)
Supplement: Supplementary file 1 — Table S1. The comparison of characteristics between the study sample and cases lost to follow‐up Table S2. The univariate analysis of risk factors for common clinical sequelae of COVID‐19 survivors. Table S3. The collinearity analysis of all covariates in the multivariate logistic regression [file IRV-16-395-s002.docx]

**Supplementary Table S1. The comparison of characteristics between the study sample and cases lost to follow-up**

|  | Study sample  (n=715) | Cases lost to follow-up  (n=236) | Statistics | *P* value |
| --- | --- | --- | --- | --- |
| Age (mean ± SD) | 70.34±6.07 | 69.94±6.65 | t=0.852 | 0.394 |
| Sex (male), n (%) | 367 (51.3%) | 120 (50.8%) | χ^2^=0.016 | 0.898 |
| Disease severity, n (%) |  |  | χ^2^=6.096 | 0.107 |
| Mild | 541 (75.7%) | 162 (68.6%) |  |  |
| Moderate | 95 (13.3%) | 45 (19.1%) |  |  |
| Severe | 48 (6.7%) | 20 (8.5%) |  |  |
| Critical | 31 (4.3%) | 9 (3.8%) |  |  |
| Comorbidity, n (%) |  |  |  |  |
| Hypertension | 277 (38.7%) | 81 (34.3%) | χ^2^=1.476 | 0.224 |
| Diabetes | 165 (23.1%) | 54 (22.9%) | χ^2^=0.004 | 0.951 |
| Hyperlipidemia | 83 (11.6%) | 28 (11.9%) | χ^2^=0.011 | 0.915 |
| Stroke | 31 (4.3%) | 11 (4.7%) | χ^2^=0.044 | 0.833 |
| Coronary heart disease | 77 (10.8%) | 28 (11.9%) | χ^2^=0.217 | 0.642 |

SD, standard deviation

**Supplementary Table S2. The univariate analysis of risk factors for common clinical sequelae of COVID-19 survivors.**

|  | Fatigue | | |  | Respiratory sequelae | | |  | Cardiovascular sequelae | | |
| --- | --- | --- | --- | --- | --- | --- | --- | --- | --- | --- | --- |
|  | Yes (n=137) | No (n=578) | *P* values |  | Yes (n=162) | No (n=553) | *P* values |  | Yes (n=36) | No (n=679) | *P* values |
| Age (years) |  |  | 0.052 |  |  |  | 0.991 |  |  |  | <0.01 |
| 55-64 | 28 (20.4%) | 72 (12.5%) |  |  | 23 (14.2%) | 77 (13.9%) |  |  | 13 (36.1%) | 87 (12.8%) |  |
| 65-74 | 82 (59.9%) | 385 (66.6%) |  |  | 106 (65.4%) | 361 (65.3%) |  |  | 10 (27.8%) | 457 (67.3%) |  |
| >74 | 27 (19.7%) | 121 (20.9%) |  |  | 33 (20.4%) | 115 (20.8%) |  |  | 13 (36.1%) | 135 (19.9%) |  |
| Sex |  |  | 0.749 |  |  |  | 0.978 |  |  |  | 0.858 |
| Male | 72 (52.6%) | 295 (51.0%) |  |  | 83 (51.2%) | 284 (51.4%) |  |  | 19 (52.8%) | 348 (51.3%) |  |
| Female | 65 (47.4%) | 283 (49.0%) |  |  | 79 (48.8%) | 269 (48.6%) |  |  | 17 (47.2%) | 331 (48.7%) |  |
| Disease severity |  |  | <0.01 |  |  |  | <0.01 |  |  |  | <0.01 |
| Mild | 82 (59.9%) | 459 (79.5%) |  |  | 93 (57.4%) | 448 (81.0%) |  |  | 15 (41.7%) | 526 (77.5%) |  |
| Moderate | 22 (16.1%) | 73 (12.6%) |  |  | 28 (17.3%) | 67 (12.1%) |  |  | 8 (22.2%) | 87 (12.8%) |  |
| Severe | 16 (11.6%) | 32 (5.5%) |  |  | 21 (13.0%) | 27 (4.9%) |  |  | 7 (19.4%) | 41 (6.0%) |  |
| Critical | 17 (12.4%) | 14 (2.4%) |  |  | 20 (12.3%) | 11 (2.0%) |  |  | 6 (16.7%) | 25 (3.7%) |  |
| Comorbidity |  |  |  |  |  |  |  |  |  |  |  |
| Hypertension | 74 (54.0%) | 203 (35.1%) | <0.01 |  | 88 (54.3%) | 189 (34.2%) | <0.01 |  | 18 (50.0%) | 259 (38.1%) | 0.155 |
| Diabetes | 45 (32.8%) | 120 (20.8%) | <0.01 |  | 52 (32.1%) | 113 (20.4%) | <0.01 |  | 13 (36.1%) | 152 (22.4%) | 0.057 |
| Hyperlipidemia | 23 (16.8%) | 60 (10.4%) | 0.035 |  | 31 (19.1%) | 52 (9.4%) | <0.01 |  | 3 (8.3%) | 80 (11.8%) | 0.789 |
| Stroke | 7 (5.1%) | 24 (4.2%) | 0.621 |  | 9 (5.6%) | 22 (4.0%) | 0.386 |  | 3 (8.3%) | 28 (4.1%) | 0.201 |
| Coronary heart disease | 22 (16.1%) | 55 (9.5%) | 0.026 |  | 25 (15.4%) | 52 (9.4%) | 0.029 |  | 8 (22.2%) | 69 (10.2%) | 0.046 |
| Treatment during hospitalization |  |  |  |  |  |  |  |  |  |  |  |
| High-flow oxygen therapy | 58 (42.3%) | 140 (24.2%) | <0.01 |  | 72 (44.4%) | 126 (22.8%) | <0.01 |  | 10 (27.8%) | 188 (27.7%) | 0.991 |
| Mechanical ventilation | 36 (26.3%) | 26 (4.5%) | <0.01 |  | 41 (25.3%) | 21 (3.8%) | <0.01 |  | 6 (16.7%) | 56 (8.2%) | 0.117 |
| Admission to ICU | 24 (17.5%) | 21 (3.6%) | <0.01 |  | 27 (16.7%) | 18 (3.3%) | <0.01 |  | 5 (13.9%) | 40 (5.9%) | 0.068 |
| Re-admission after discharge | 25 (18.2%) | 23 (4.0%) | <0.01 |  | 30 (18.5%) | 18 (3.3%) | <0.01 |  | 7 (19.4%) | 41 (6.0%) | 0.007 |

Data are n (%). *P* values were calculated by χ² test or Fisher’s exact test as appropriate.

**Supplementary Table S3. The collinearity analysis of all covariates in the multivariate logistic regression**

| Covariates | Collinearity Statistics | |
| --- | --- | --- |
|  | Tolerance | Variance Inflation Factor |
| Age | 0.970 | 1.031 |
| Sex | 0.978 | 1.022 |
| Hypertension | 0.852 | 1.174 |
| Diabetes | 0.800 | 1.249 |
| Hyperlipidemia | 0.837 | 1.195 |
| Stroke | 0.928 | 1.077 |
| Coronary Heart Disease | 0.874 | 1.145 |
| Disease severity | 0.672 | 1.487 |
| High-flow oxygen therapy | 0.779 | 1.283 |
| Mechanical ventilation | 0.429 | 2.334 |
| Admission to ICU | 0.445 | 2.246 |
| Re-admission after discharge | 0.903 | 1.107 |
