# Supplementary figures and images for: Some characteristics of clinical sequelae of COVID‐19 survivors from Wuhan, China: A multi‐center longitudinal study
Source: Influenza Other Respir Viruses. 2021 Nov 19;16(3):395–401. doi: 10.1111/irv.12943 (PMC8652839; doi:10.1111/irv.12943)

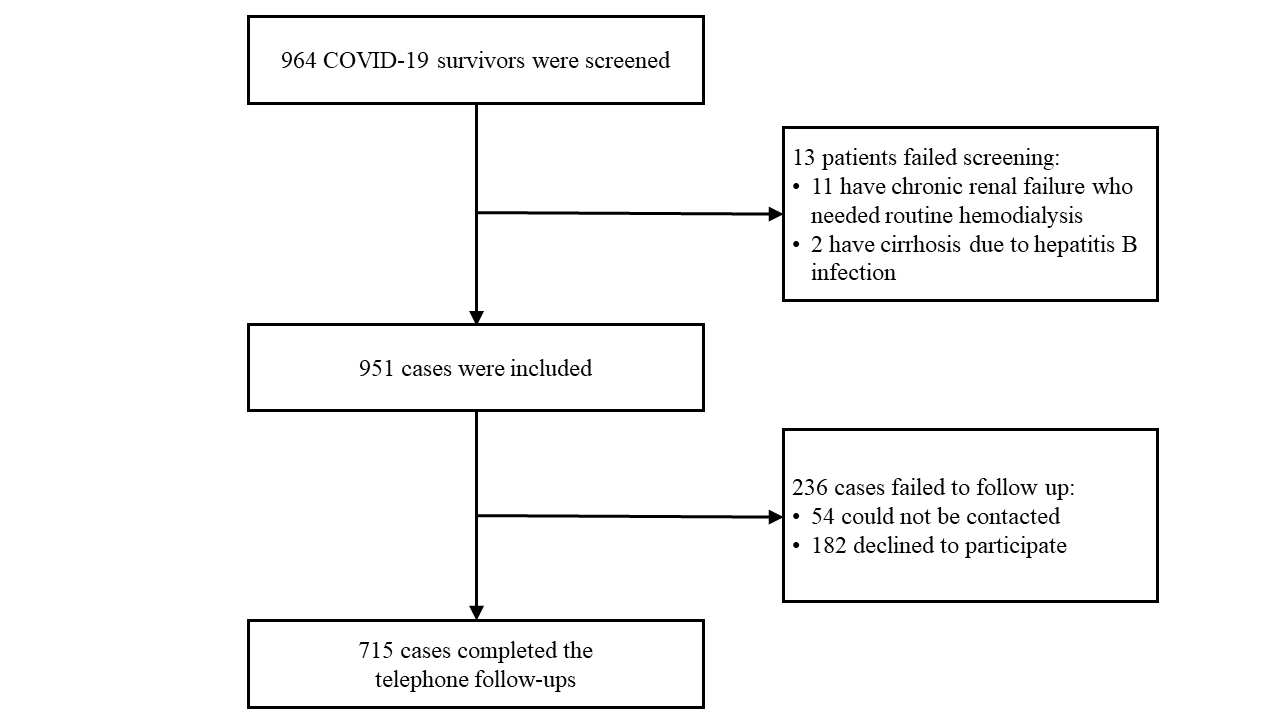

Supplement: Supplementary file 2 — Figure S1. The flow chart of the study sample [file IRV-16-395-s001.tif]
